# Synthesis, Crystal Structure and Thermoelectric Properties of the Type-I Clathrate Sn_38_Sb_8_I_8_

**DOI:** 10.3390/nano15221727

**Published:** 2025-11-16

**Authors:** Nikolaos Moutzouris, Panagiotis Mangelis, Nikolaos Kelaidis, Nagia S. Tagiara, Emmanuel Klontzas, Ioannis Koutselas, Panagiotis Oikonomopoulos, Themistoklis Sfetsas, Theodora Kyratsi, Andreas Kaltzoglou

**Affiliations:** 1 Theoretical and Physical Chemistry Institute, National Hellenic Research Foundation, 11635 Athens, Greece; nmoutz@eie.gr (N.M.); nkelaidis@eie.gr (N.K.); ntayara@eie.gr (N.S.T.); klontzas@eie.gr (E.K.); 2 Department of Mechanical Engineering, University of Cyprus, 1678 Nicosia, Cyprus; mangelis.panagiotis@ucy.ac.cy (P.M.); kyratsi.theodora@ucy.ac.cy (T.K.); 3 Materials Science Department, School of Natural Sciences, University of Patras, 26504 Rio, Greece; ikouts@upatras.gr; 4 Department of Chemistry, National and Kapodistrian University of Athens, 15772 Athens, Greece; poikon@chem.uoa.gr; 5 Research & Development, Quality Control and Testing Services, QLAB Private Company, 57008 Thessaloniki, Greece; tsfetsas@q-lab.gr

**Keywords:** host–guest interactions, Zintl–Klemm concept, thermoelectric properties, Raman spectroscopy

## Abstract

Semiconducting clathrates are a distinct class of inclusion compounds with considerable interest for thermoelectric applications. We report here the synthesis, crystal structure and thermoelectric properties of Sn_38_Sb_8_I_8_. The compound was synthesized via planetary ball milling of the corresponding elements for 6 h and then sintering of amorphous mixture at 620 K for 3 days. The crystal structure of the polycrystalline product was determined via X-ray powder diffraction and Rietveld refinement as a type-I clathrate (*a* = 12.0390(2), space group *Pm*-3*n*, No. 223) with mixed-occupied Sn/Sb framework sites and fully occupied I guest sites. Further analysis on the chemical composition, nanomorphology and vibrational modes of the material was carried out via Induced-Coupled-Plasma–Mass Spectrometry, SEM/EDX microscopy and Raman spectroscopy, respectively. Thermoelectric measurements were performed on hot-pressed samples with ca. 98% of the crystallographic density. The clathrate compound behaves as an *n*-type semiconductor with a band gap of 0.737 eV and exhibits a maximum ZT of 0.0016 at 473 K. Theoretical calculations on the formation enthalpy, electron density of states and transport properties provide insights into the experimentally observed physical behavior.

## 1. Introduction

Clathrate compounds are a class of inclusion materials in which guest atoms or molecules are completely and irreversibly encapsulated within the cavities of a host framework [1]. Depending on the chemical composition of this framework, they are broadly categorized into clathrate hydrates, where the framework is built of water molecules, and ‘intermetallic’ or ‘semiconducting’ clathrates, which consist primarily of four-bonded, group-fourteen elements (Si, Ge, Sn) [2]. Intermetallic clathrates can further be classified based on the framework’s charge, leading to polyanionic clathrates which incorporate electropositive guest atoms (e.g., alkali or alkaline-earth metals), and polycationic or inverse clathrates where the framework carries a positive charge and halogen atoms (I, Br, Cl) or Te atoms serve as guests [3,4]. Their exact composition is often described via the electron-counting rules of the Zintl–Klemm concept [5].

**Figure 1:**
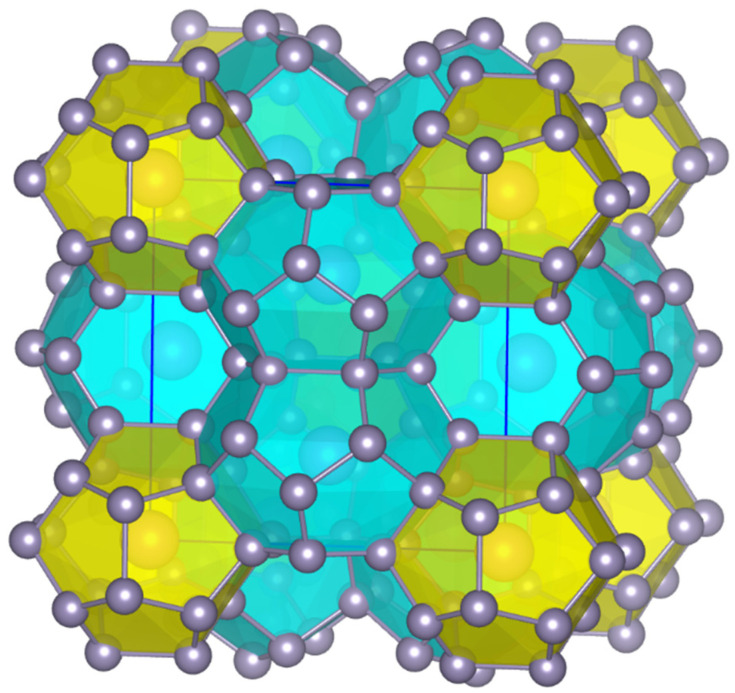
Crystal structure of Sn_38_Sb_8_I_8_ clathrate with mixed-occupied Sn/Sb framework sites. I atoms reside in the center of the pentagonal dodecahedra (yellow) and the tetrakaidecahedra (light blue). The illustration of the crystal structure was performed using the VESTA software version 3.90.1 [16].

Compared to their polyanionic counterparts, the polycationic clathrates are far less explored. They are usually synthesized from the corresponding elements either by ball milling and sintering or by hot pressing and contain indium, phosphorus, antimony and zinc for framework substitution [6,7,8,9,10,11,12,13,14,15]. In terms of physical properties, polycationic clathrates exhibit so far moderate thermoelectric efficiency [3]. With regard to Sn-based clathrates, Sn_38_Sb_8_I_8_ has been initially reported by Kishimoto et al. [6], which adopts the type-I clathrate structure, with nominal charges (4b-Sn^0^)_38_(4b-Sb^+^)_8_I^−^_8_ (Figure 1) and exhibits semiconducting behavior. We hereby perform an integrated study on the synthesis, chemical composition, vibrational spectroscopy, DFT calculations and thermoelectric properties of the type-I clathrate Sn_38_Sb_8_I_8_.

## 2. Materials and Methods

The reagents Sn (powder, 99.999%), Sb (powder, 99.999%) and I (grains, 99.999%) were obtained by Sigma-Aldrich (United States) and were initially ground together in an atomic ratio of 38:8:8, respectively, in an agate mortar. The mixture (ca. 3 g per batch) was then loaded into a Retsch PM100 planetary ball mill (Rastatt, Germany), inside a 25 mL stainless steel jar along with 5 mm diameter stainless steel balls and spun at 600 rpm for 6 h. The obtained dark gray powder was subsequently loaded in a pyrex tube that was sealed under vacuum (∼0.1 mbar) and heated to 623 K for 3 days. The resulting crystalline gray powder was air stable and was used for all thermoelectric measurements described below without any purification. Moreover, the same process was repeated for a Sn:Sb:I atomic ratio of 30:16:8 and it is discussed separately with regard to the homogeneity range in this ternary system.

The reaction products were structurally characterized by PXRD using a Bruker D8-Advance diffractometer (Karlsruhe, Germany) with Lynx-Eye XE-T silicon strip detector in Bragg−Brentano geometry with Cu-Kα_1_ (*λ* = 1.5406 Å) and Cu-Kα_2_ (*λ* = 1.5444 Å) radiation. Diffraction data were collected over the range 5° ≤ 2θ ≤ 80° counting for 1 s at each step of 0.02° in the detector position. Rietveld refinements were carried out using the FULLPROF software version 5.20 [17]. The parameters refined with the least-squares method are the sample displacement, the scale factor and the lattice parameters for each phase, the atomic coordinates (paired for every Sn/Sb position) for general positions, the isotropic displacement parameters (common for every Sn/Sb pair) for all atoms in the clathrate structure and some peak profile parameters. The background is fitted via linear interpolation for a set of refinable points.

SEM/EDX measurements were performed on a Zeiss EVO-MA10 (Oberkochen, Germany), equipped with an Oxford EDS analyzer, where the electron beam accelerating voltages used were between 5 and 12 kV with currents of 270 pA for imaging and up to 2 nA for EDX measurements. Charge accumulation was avoided by fast interlaced scanning and averaging techniques.

IPC-MS measurements were performed on an Agilent Technologies Model 7850 instrument (Agilent Technologies, Santa Clara, CA, USA) equipped with the ORS4 collision cell for the analysis of macro-elements and trace metals. The solid samples were dissolved in aqua regia and diluted in deionized water. Sampling was performed using an Agilent SPS 4 autosampler. The 7850 ICP-MS was configured with the standard ISIS 3 injection system. The IntelliQuant function in the ICPMS MassHunter 5.1 software provides the capability of a full mass-spectrum scan with only two seconds additional measurement time, though the samples were quantitated by internal standard seven-point calibration.

Raman spectroscopy was carried out under backscattering geometry on a Renishaw inVia Raman Microscope (Charfield, United Kingdom), equipped with a 2400 line/mm diffraction grating, a high sensitivity Peltier-cooled charged couple device (CCD), a motorized xyz microscope stage, a 50x magnification lens and a Rayleigh rejection notch filter, allowing for measurements down to 5 cm^−1^. All measurements were made at room temperature with a 2 cm^−1^ resolution using a 514.5 nm Ar ion laser for excitation employing ca. 0.10 mW μm^−2^ on the sample with no signs of laser-induced modifications observed. Each spectrum is an average of 35 scans in the range of 5–300 cm^−1^ accumulated for about 20 min to improve the signal-to-noise ratio.

Uniaxial hot press sintering was performed on the polycrystalline powders at 673 K and 80 MPa for 1 h under Ar atmosphere in a HP20, Thermal Technologies system. The thermal diffusivity (*D*) of the sample was measured by a Netzsch LFA 457 laser setup (Selb, Germany). Data were collected in 25 K increments on pellet coated with graphite over the temperature range 300–473 K. The determination of specific heat capacity (*Cp*) was performed based on the comparative method using a pyroceram sample as reference. The thermal conductivity was determined by using the formula:

*κ* = *D*
*ρ*
*Cp* (1)

Electrical resistivity and Seebeck coefficient measurements were performed using a ZEM-3 ULVAC-RIKO (Japan) instrument over the temperature range 293–473 K. Estimated uncertainties for the measurements of electrical and thermal transport properties are ±5% and ±7%, respectively.

The DFT calculations were performed using the Vienna Ab initio Simulation Package (VASP, vs. 6) [18,19,20]. The exchange–correlation interactions were treated using the Perdew–Burke–Ernzerhof functional revised for solids (PBESOL), along with the soft pseudopotentials provided by VASP. Dispersion interactions were included by using the semi-empirical DFT-D2 method of Grimme [21], which has been shown to improve structural accuracy in van der Waals and guest–host systems. The plane-wave cutoff energy was set at 520 eV and the Brillouin zone was sampled with a 4 × 4 × 4 Monkhorst-Pack k-point grid. The self-consistent field (SCF) convergence criterion was set at 1.0 × 10^−8^ eV/atom, whereas the force tolerance for ionic relaxations was 0.01 eV/Å. A structural model was used without Sb- and Sn-mixed occupancies in the space group *Pm*-3 (No. 200), where the Sb atoms are fully occupied on the 8i Wyckoff site, located in pentagonal rings of the tetrakaidecahedra. This is a maximal translation-equivalent subgroup of the experimentally determined space group *Pm*-3*n* (No. 223). In all structural optimization attempts, both atomic coordinates and cell parameters were allowed to relax, with no symmetry constraints imposed. The conjugate gradient minimization scheme was used to optimize energies and forces. To assess the thermodynamic stability of the compound, the simplified formula for the formation enthalpy was used:

(2) $$\Delta Hf=E_{tot}-\sum_{i}{n_{i}\mu }_{i}$$

where *E_tot_* is the total energy and *μ_i_* the chemical potential of each element at its most stable form. For the Density of States (DOS) calculations, a denser 8 × 8 × 8 k-point grid was employed and the resulting spectra were analyzed with the SUMO toolkit [22]. Transport coefficients were investigated using the semiclassical Boltzmann transport theory as implemented in BoltzTraP2 code (Wien, Austria) [23]. In addition, the influence of carrier doping was probed to simulate the impact of unintentional impurities or charge transfer at contacts.

## 3. Results and Discussion

### 3.1. Structural and Chemical Analysis

**Figure 2:**
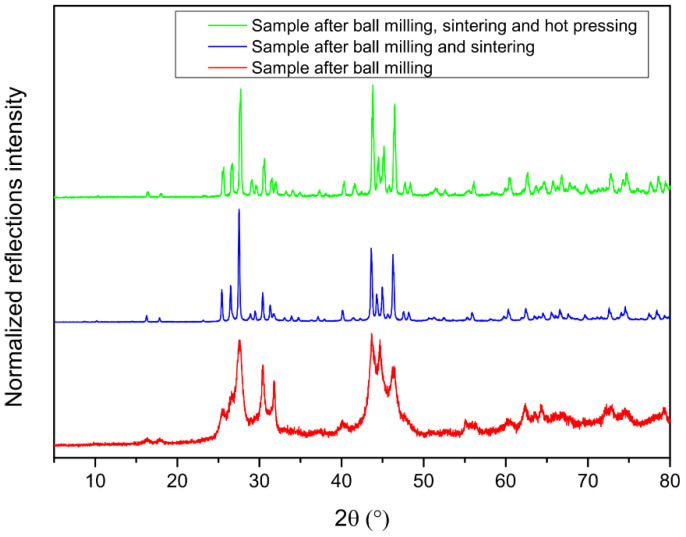
PXRD patterns for different steps of the Sn_38_Sb_8_I_8_ clathrate synthesis.

The X-ray powder diffraction data are shown in Figure 2. The formation of the clathrate phase takes place gradually and in low crystallinity upon ball milling for 6 h (Table 1). The sintering process leads to an almost pure phase sample, with minor impurities of β-Sn and a 1:1 Sn-Sb alloy (space group *R*-3*m*, No. 166, *a* = *b* = 4.3282(1) Å, *c* = 5.3500(1) Å) (Figure S1). No sign of underoccupied sites or superstructure reflections is observed through the structural refinements (Table S1), in contrast to other polycationic clathrates, e.g., Sn_24_P_19.3_I_8_ [14] and to polyanionic clathrates, e.g., Cs_8_Sn_44_ [24,25] which often have defect framework sites in order to fulfill the Zintl–Klemm concept, as well as lower symmetry due to defect ordering than the archetype structure. As expected from host–guest distances that range between 3.79 and 4.69 Å, the isotropic thermal displacement values of the I atoms are significantly larger (almost double in the case of the tetrakaidecahedron) compared to the framework atoms, due to their loose bonds.

The SEM/EDX analysis reveals that the sample after ball milling contains large amounts of Sn/Sb alloy in the form of small spherical particles (ca. 2 μm diameter) as well as a bulkier material with variable ratios of Sn, Sb and I atoms (Figure S2). After ball milling and sintering the sample becomes more homogeneous and the composition of the bulk phase is close to the anticipated 38:8:8 ratio for Sn, Sb and I atoms, respectively. On the other hand, the ICP-MS analysis has shown that the samples contained traces of Fe stemming from the ball mill jar and balls, during the synthesis. This amount is in ppm level and does not seem to have a significant effect on the thermoelectric properties of the sample. With regard to the homogeneity range in this system, the 30:16:8 reaction stoichiometry led to the PXRD pattern shown in Figure S3. Large amount of unreacted Sn–Sb alloy was detected via XRPD (Table 1). A significant reduction in the lattice parameters is found for the Sb-rich clathrates by ca. 0.1 Å, which shows that the electron count is lower than in the 38:8:8 stoichiometry. This leads to the conclusion that the actual Sb content in this clathrate is between the ratios 38:8:8 and 30:16:8. Due to the large concentration of metallic impurities, this material was not studied further for its thermoelectric properties.

### 3.2. Raman Spectroscopy

**Figure 3:**
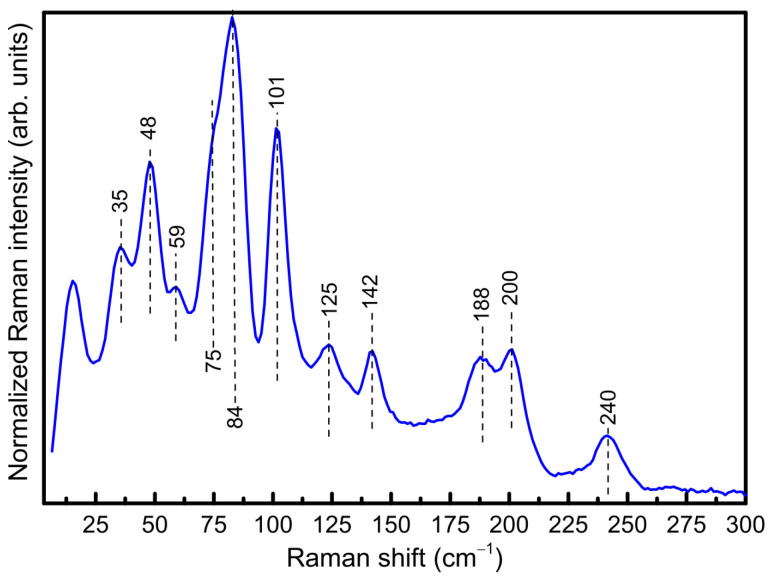
Raman spectrum of the ball-milled and sintered Sn_38_Sb_8_I_8_ clathrate.

The room temperature Raman spectrum of Sn_38_Sb_8_I_8_ is shown in Figure 3. Similarly to previous studies [26,27], the low-energy peaks, characteristic of clathrate structures with restricted guest motions (known as “rattling modes”) are observed at ca. 35 cm^−1^. This is closely related to the large thermal displacement values determined for the iodine atoms via Rietveld refinement, as mentioned above. At higher frequencies, the peaks are attributed to the framework vibrations. In particular, the presence of Sb atoms leads to many distinct vibrational modes around 100–150 cm^−1^ due to its different mass and chemical bonding compared to Sn.

### 3.3. Thermoelectric Properties

**Figure 4:**
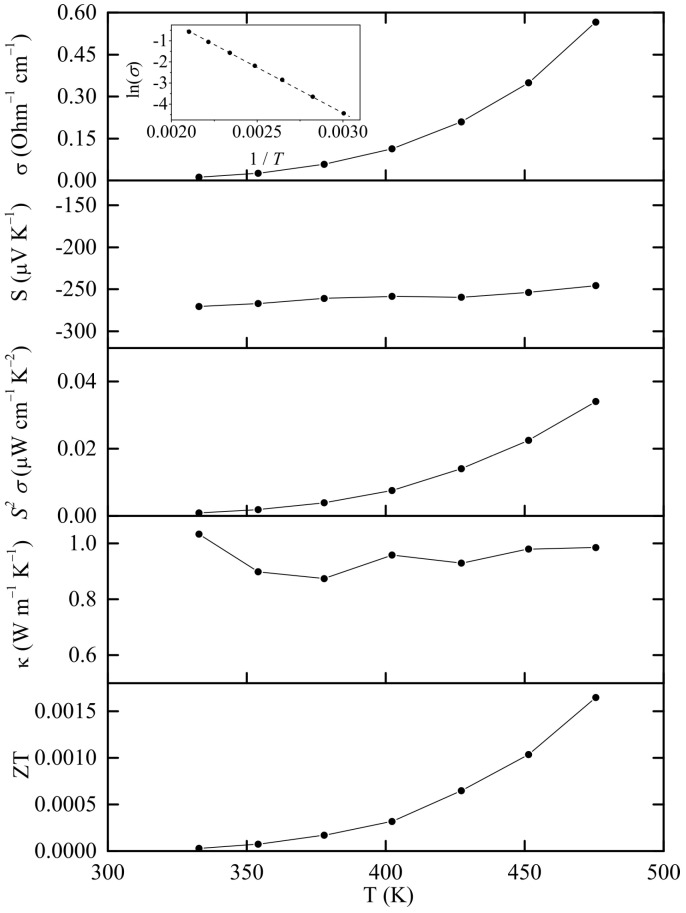
Experimental thermoelectric properties of Sn_38_Sb_8_I_8_. The inset shows the Arrhenius fit of the electrical conductivity values as a function of temperature in order to determine the electronic band gap.

The density of the hot-pressed pellet was ρ = 6.07 g cm^−3^, which corresponds to ca. 98% of the crystallographic value. The thermoelectric properties of Sn_38_Sb_8_I_8_ are given in Figure 4. The sample shows a semiconducting behavior with exponential electrical conductivity increase over temperature. According to the Arrhenius equation from the electrical conductivity measurements, the band gap is determined at 0.737 eV (inset in Figure 4). The Seebeck coefficient and thermal conductivity range between −245 to −270 μV K^−1^ and 0.87 to 1.03 W m^−1^ K^−1^, respectively. To our knowledge, the only previous experimental study [6] on this material shows electrical conductivity of 10^−3^ Ohm^−1^ cm^−1^ at RT and 0.1 10^−3^ Ohm^−1^ cm^−1^ at 500 K, Seebeck coefficient of −600 μV K^−1^ at 300 K and −500 μV K^−1^ at 550 K, an almost constant thermal conductivity of 0.7 W m^−1^ K^−1^ and a band gap of 0.8 eV. The main reason for the deviation with our results may be the partial decomposition of the sample during the hot-pressing process. As shown in Table 1 and Figure S4, the clathrate compound has decomposed by ca. 18% into *β*-Sn and Sn–Sb alloy which show more metallic behavior, which increases the electrical conductivity and decreases in the Seebeck coefficient in absolute value.

### 3.4. Computational Analysis

#### 3.4.1. Structural Optimization and Thermodynamic Stability

The initial structural model was fully relaxed until both atomic positions and cell parameters reached equilibrium. Using the standard PBEsol functional, the optimized lattice constant was found to be 12.128 Å, which is in moderate agreement with our current experimental data (12.0390(2) Å) or the previous literature (e.g., 12.0447(3) Å [3]). When including the dispersion corrections using the PBEsol+D2 method, the lattice constant was calculated at 12.033 Å, essentially reproducing the experimental value with excellent accuracy. This confirms that long-range van der Waals interactions between the I guest atoms and the Sb/Sn framework are important in stabilizing the clathrate structure. The PBEsol+D2 geometry was therefore adopted for all subsequent electronic structure and transport calculations. Guest atoms were accommodated without off-centering in the dodecahedral and tetrakaidecahedral cages. To assess stability, the formation enthalpy was calculated at negative values of −15.98 eV per formula unit (which corresponds to −0.2854 eV per atom), which confirms the thermodynamic stability of the compound relative to its components (at their most stable form).

#### 3.4.2. Electronic Structure: Density of States

**Figure 5:**
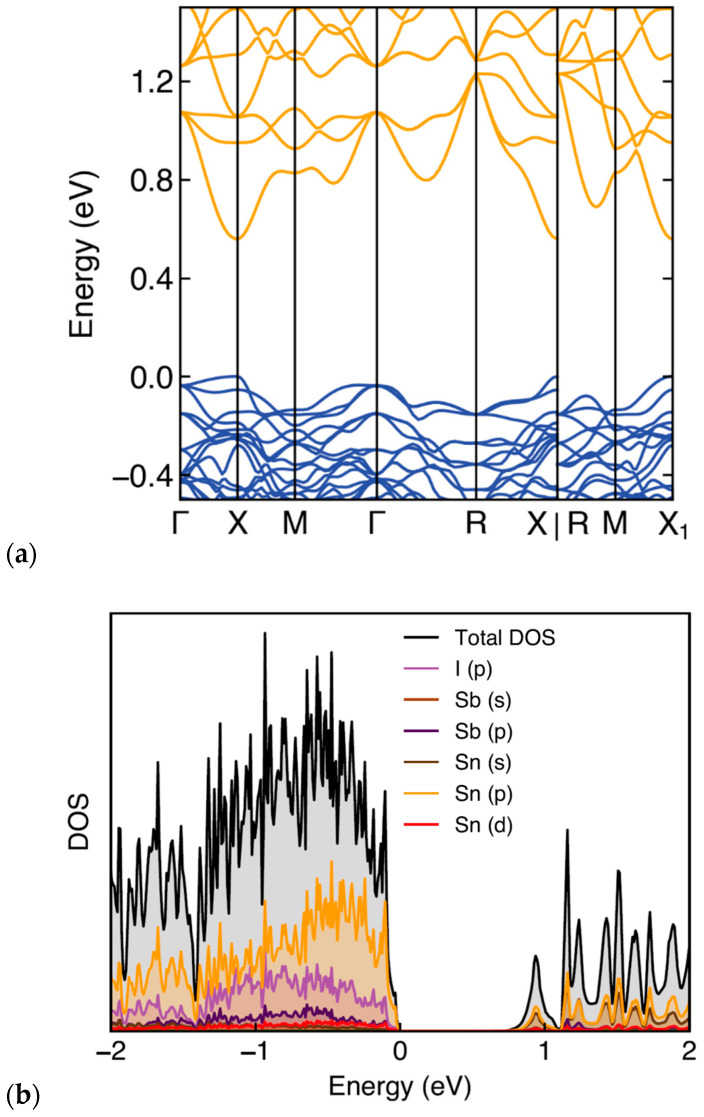
(**a**) Electronic band structure of Sb_8_Sn_38_I_8_ along the high-symmetry directions in the Brillouin zone. (**b**) Total and projected density of states (DOS).

The calculated electron band structure and the corresponding total and projected electronic density of states (DOS) of Sn_38_Sb_8_I_8_ are shown in Figure 5, where the Fermi level is set at 0 eV. The band structure indicates that the system exhibits a semiconducting behavior, with a direct energy band gap at the X-point in the Brillouin k-space. DOS calculations show a band gap of 0.71 eV for Sb_8_Sn_38_I_8_. This result is close to the experimental value of 0.737 eV but significantly larger than 0.24 eV reported by Eto et al. [13], likely reflecting improved structural relaxation and basis set convergence compared with less modern calculations. The projected DOS (Figure 5b) shows that the valence band is primarily composed of Sn(p) and I(p) states, with smaller contributions from Sb(p), while the conduction band is dominated by Sn s and p orbitals. The pronounced iodine p-character near the valence-band edge confirms strong guest–host hybridization, which produces the sharp DOS features also reported in [13]. This hybridization gives rise to mixed ionic–covalent bonding between the Sn/Sb framework and the iodine guests, stabilizing the structure and accords with the Zintl–Klemm concept.

#### 3.4.3. Transport Properties

We apply the Boltzmann transport equation to calculate the Seebeck coefficient S(T) and the conductivity prefactor σ(Τ)/τ, within the constant relaxation time approximation. Under this condition, the electron relaxation time is assumed to be independent of temperature, band index, and wave vector, simplifying the solution of the Boltzmann equation. Transport coefficients were computed using BoltzTraP2 code, based on the previously converged DFT band structure. The band interpolation was performed with a dense Fourier mesh and an energy window of ±0.5 eV around the Fermi level.

**Figure 6:**
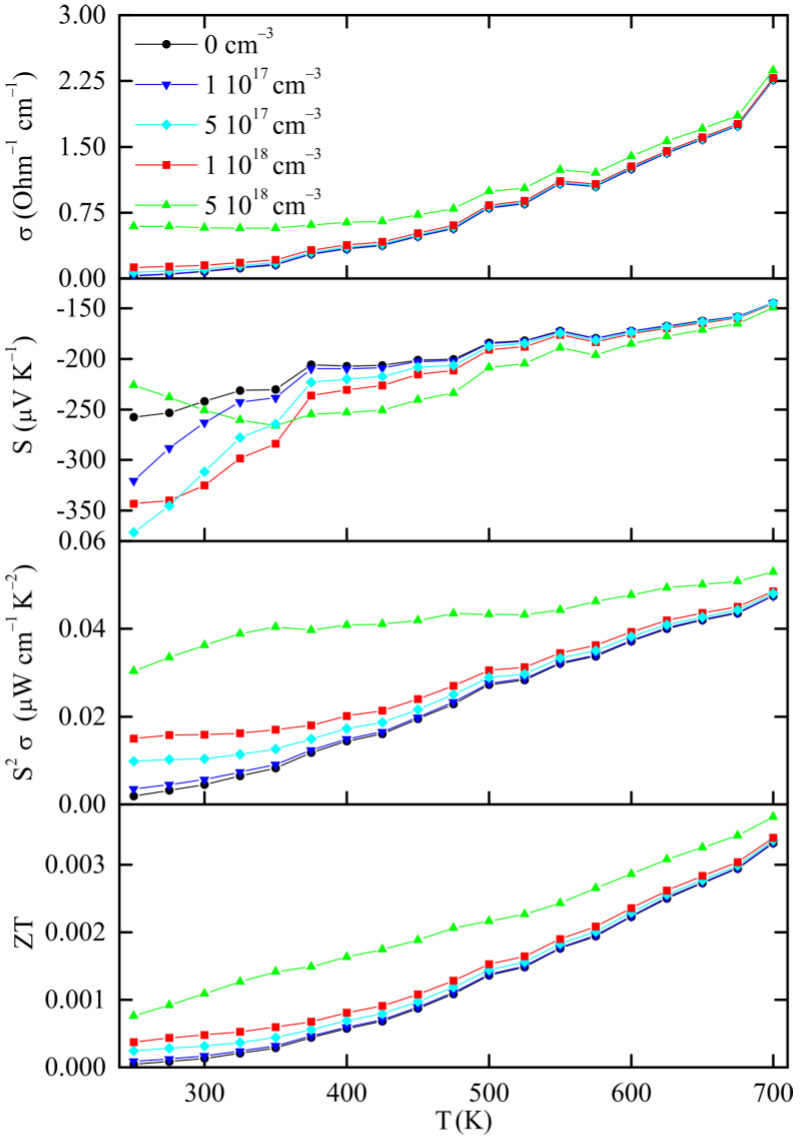
Calculated thermoelectric properties of Sb_8_Sn_38_I_8_ after DFT relaxation.

We calculated the electrical conductivity, Seebeck coefficient and power factor (S^2^σ), as functions of carrier concentration and temperature. The compound is intrinsically *n*-type, with the Fermi level located near the conduction-band minimum. Calculations were performed for electron concentrations between 1 × 10^17^ cm^−3^ and 1 × 10^19^ cm^−3^ under the constant relaxation time approximation, corresponding to different positions of the chemical potential within the conduction band. Typical values of tau (τ) in the literature are τ = 1 × 10^−14^ s or less [28,29]. Here, we apply the value of τ = 1 × 10^−16^ s to obtain calculations of electrical conductivity and power factor in the same order of magnitude with the experimental results, as shown in Figure 6.

The *σ* curves show two regimes characteristic of *n*-type semiconductors. At lower carrier concentrations, *σ* and *σ*/*τ* increase rapidly with temperature, reflecting a thermally activated transport mechanism. In contrast, for higher concentrations, it remains stable or decreases slightly for the high doping cases. It should be noted that the constant relaxation time model does not explicitly include temperature-dependent scattering mechanisms such as electron–phonon or impurity scattering. The observed mild decrease or U-shaped behavior of σ(T) at high carrier concentrations is therefore due to band-structure effects—mainly the downward shift in the chemical potential and the thermal broadening of the Fermi–Dirac distribution, which reduce the effective carrier velocity at elevated temperatures.

The calculated Seebeck coefficients are negative throughout the investigated range, confirming the *n*-type character of charge transport. At 300 K, |S| varies from approximately −360 µV K^−1^ at low electron concentrations (~5 × 10^17^ cm^−3^) to −200 µV K^−1^ at higher concentrations (~5 × 10^18^ cm^−3^). As temperature increases, |S| gradually decreases for highly doped cases but remains nearly constant or slightly increases for the lower carrier concentrations, indicating a crossover from degenerate to non-degenerate behavior at elevated temperatures.

The calculated power factor exhibits a maximum at high electron concentrations (~10^18^ cm^−3^) at temperatures near 700 K and reaches a maximum of approx. 6 µW m^−1^ K^−2^, where the opposing trends of decreasing Seebeck coefficient and increasing electrical conductivity are balanced. We note here that in our calculations we used a relaxation time value of τ = 10^−16^ s to approximately match the experimentally observed conductivity of iodine-filled clathrates. This is shorter than typical relaxation times (usually around 10^−14^–10^−15^ s) and is attributed to the strong scattering of charge carriers caused by the heavy iodine atoms and the mixed Sb/Sn framework. The high resistivity observed experimentally for Sb_8_Sn_38_I_8_ [13] supports this hypothesis. It is also noted that changing τ only scales the absolute values of σ but it does not affect the Seebeck coefficient or its temperature dependence, which are determined by the underlying band structure.

The overall behavior of σ(T) and S(T) in our results follows the typical trends observed in other semiconducting thermoelectrics. The moderate decrease in |S| with temperature and its dependence on carrier concentration are consistent with the *n*-type response predicted for doped SnSe and SnS by [29]. Likewise, the broad maximum in power factor around 500 K mirrors that seen in Sb-based clathrates such as Ba_8_Ga_16_Sn_30_ and in the iodine-doped Sn systems studied by [13]. The results indicate that charge transport in Sb_8_Sn_38_I_8_ is dominated by intrinsic band-structure features, particularly the weakly dispersive iodine-derived states near the band edges. This intrinsic semiconducting behavior leads to limited carrier mobility and moderate power factors.

The calculated ZT versus temperature for Sb_8_Sn_38_I_8_ is shown in Figure 6, where a simplified constant lattice thermal conductivity of κ_L_ = 1 W m^−1^ K^−1^ was presumed. It is calculated that higher carrier concentrations yield larger ZT values, primarily due to enhanced electrical conductivity. At 300 K, ZT values remain close or below 0.001 for all doping levels but increase steadily to reach approximately 0.003 at 700 K for the most highly doped case (n ≈ 5 × 10^18^ cm^−3^). At low and intermediate doping levels, we observe nearly parallel curves, consistent with the semiconducting nature of this compound. These computed ZT values match closely the experimental measurements (ZT ≈ 0.0016 at 473 K), confirming that the low performance arises mainly from limited carrier mobility and intrinsic scattering. Nevertheless, the present calculations are an estimation of the potential thermoelectric performance achievable in Sn_38_Sb_8_I_8_ upon controlled *n*-type doping. Optimization of the carrier concentration could significantly enhance its efficiency while retaining the low lattice thermal conductivity characteristic of clathrates [30]. In a broader context, many Sn-based materials have gained attention in the past years, such as SnO_2_ [31], SnTe [32] and type-VIII clathrate Ba_8_Ga_16_Sn_30_ [33], as they exhibit high thermoelectric efficiency combined with the inherent low cost of the row materials as well as ease of fabrication.

## 4. Conclusions

The title compound is synthesized in high purity in the scale of a few grams per batch. It behaves as an *n*-type semiconductor with maximum ZT of 0.0016 at 473 K. Despite the low figure of merit, this synthetic route allows for a reproducible synthesis of new polycationic clathrates with different types of framework doping. Moreover, the guest–host vibrational modes were investigated through Raman spectroscopy, revealing the characteristic low-energy rattling of the iodine atoms. The theoretical calculations predict an almost linear increase in the ZT with increasing temperatures up to 700 K. Future improvements in the thermoelectric performance may be achieved through controlled carrier doping or compositional tuning in order to reduce the electronic band gap and to enhance the carrier mobility.

## Figures and Tables

**Table 1:** Lattice parameters and quantitative analysis from XRPD and Rietveld refinement on various samples with the nominal composition Sn_38_Sb_8_I_8_ and Sn_30_Sb_16_I_8_.

Sample and Its Nominal Composition	Lattice Parameter, a (Å)	Amount of β-Sn Impurity (wt%)	Amount of Sn–Sb Alloy Impurity (wt%)
Ball-milled and sintered Sn_38_Sb_8_I_8_	12.0390(2)	3.1(1)	1.4(1)
Ball-milled, sintered and hot-pressed Sn_38_Sb_8_I_8_	12.0401(1)	12.1(4)	6.4(2)
Ball-milled and sintered Sn_30_Sb_16_I_8_	11.9486(2)	0	36.4(4)

## Data Availability

The original contributions presented in this study are included in the article/Supplementary Material. Further inquiries can be directed to the corresponding authors.

## Supplementary Materials

The following supporting information can be downloaded at https://www.mdpi.com/article/10.3390/nano15221727/s1, Figure S1: Rietveld plot for Sn_38_Sb_8_I_8_ after ball milling and sintering; Table S1: Crystallographic parameters from the Rietveld refinement for Sn_38_Sb_8_I_8_ after ball milling and sintering; Figure S2: SEM images of Sn_38_Sb_8_I_8_ samples at different steps of the synthesis; Figure S3: Rietveld plot for Sn_30_Sb_16_I_8_ after ball milling and sintering; Figure S4: Rietveld plot for Sn_38_Sb_8_I_8_ after ball milling, sintering and hot pressing.

## Abbreviations

The following abbreviations are used in this manuscript:

PXRD	Powder X-ray Diffraction
ZT	Thermoelectric figure of merit
DFT	Density Functional Theory
DOS	Density of States
ICP-MS	Induced Coupled Plasma-Mass Spectrometry
SEM	Scanning Electron Microscopy
EDX	Energy-Dispersive X-ray analysis
