# Supplementary material for: Synthesis, Crystal Structure and Thermoelectric Properties of the Type-I Clathrate Sn38Sb8I8
Source: Nanomaterials (Basel). 2025 Nov 16;15(22):1727. doi: 10.3390/nano15221727 (PMC12655030; doi:10.3390/nano15221727)
Supplement: Supplementary file 1 [file nanomaterials-15-01727-s001.zip › nanomaterials-3986754-supplementary.pdf]

Supporting Information

Synthesis, Crystal Structure and Thermoelectric Properties of the Type-I Clathrate $Sn_{38}Sb_8I_8$

Nikolaos Moutzouris [1], Panagiotis Mangelis [2], Nikolaos Kelaidis [1], Nagia S. Tagiara [1], Emmanuel Klontzas [1], Ioannis Koutselas [3], Panagiotis Oikonomopoulos [4], Themistoklis Sfetsas [5], Theodora Kyratsi [2] and Andreas Kaltzoglou [1,*]

[1] Theoretical and Physical Chemistry Institute, National Hellenic Research Foundation, 11635 Athens, Greece; nmoutz@eie.gr (N.M.); nkelaidis@eie.gr (N.K.); ntayara@eie.gr (N.S.T.); klontzas@eie.gr (E.K.)
[2] Department of Mechanical Engineering, University of Cyprus, 1678 Nicosia, Cyprus; mangelis.panagiotis@ucy.ac.cy (P.M.); kyratsi.theodora@ucy.ac.cy (T.K.)
[3] Materials Science Department, School of Natural Sciences, University of Patras, 26504 Rio, Greece; ikouts@upatras.gr
[4] Department of Chemistry, National and Kapodistrian University of Athens, 15772 Athens, Greece; poikon@chem.uoa.gr
[5] Research & Development, Quality Control and Testing Services, QLAB Private Company, 57008 Thessaloniki, Greece; tsfetsas@q-lab.gr
* Correspondence: akaltzoglou@eie.gr; Tel.: +302107273845

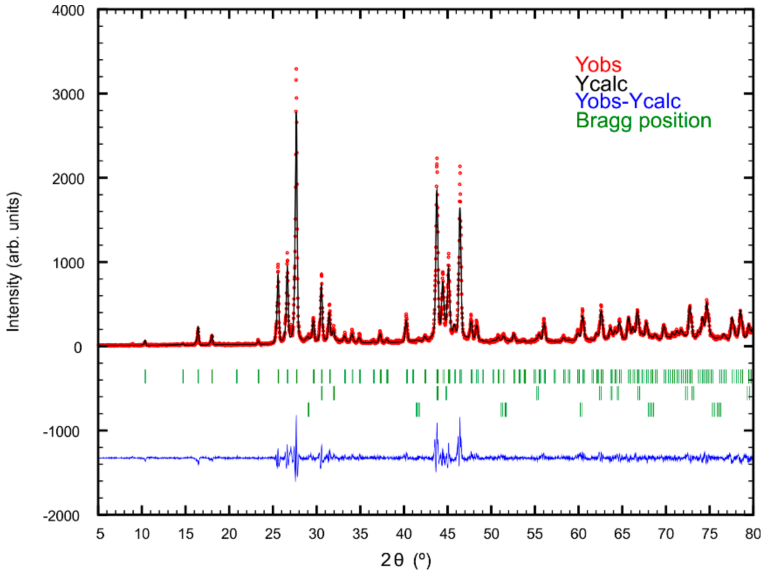

Figure S1. Rietveld plot using the powder X-ray diffraction data for the ball-milled and sintered sample of $Sn_{38}Sb_8I_8$. Residual values: R_{wp} = 18.0%, χ^2 = 3.09, $R_{Bragg}(Sn_{38}Sb_8I_8)$ = 10.3%, R_{Bragg}(β-Sn) = 11.9%, R_{Bragg}(Sn-Sb alloy) = 18.5%. The top, middle and bottom rows of Bragg peaks represent $I_8Sn_{38}Sb_8$, β-Sn and a Sb–Sn alloy, respectively.

Table S1. Wyckoff sites, fractional atomics coordinates, and isotropic thermal displacement parameters from the Rietveld refinement using the powder X-ray diffraction data for the ball-milled and sintered $Sn_{38}Sb_8I_8$ sample.

**Atom**	**Wyckoff site**	**x/a**	**y/b**	**z/c**	**U_{iso} (Å^2)**
I1	2a	0	0	0	0.028(3)
I2	6d	¼	½	0	0.041(2)
Sn1	6c	¼	0	½	0.021(2)
Sb1	6c	¼	0	½	0.021(2)
Sn2	16i	0.1830(2)	0.1830(2)	0.1830(2)	0.0194(9)
Sb2	16i	0.1830(2)	0.1830(2)	0.1830(2)	0.0194(9)
Sn3	24k	0	0.3117(2)	0.1163(2)	0.0233(8)
Sb3	24k	0	0.3117(2)	0.1163(2)	0.0233(8)

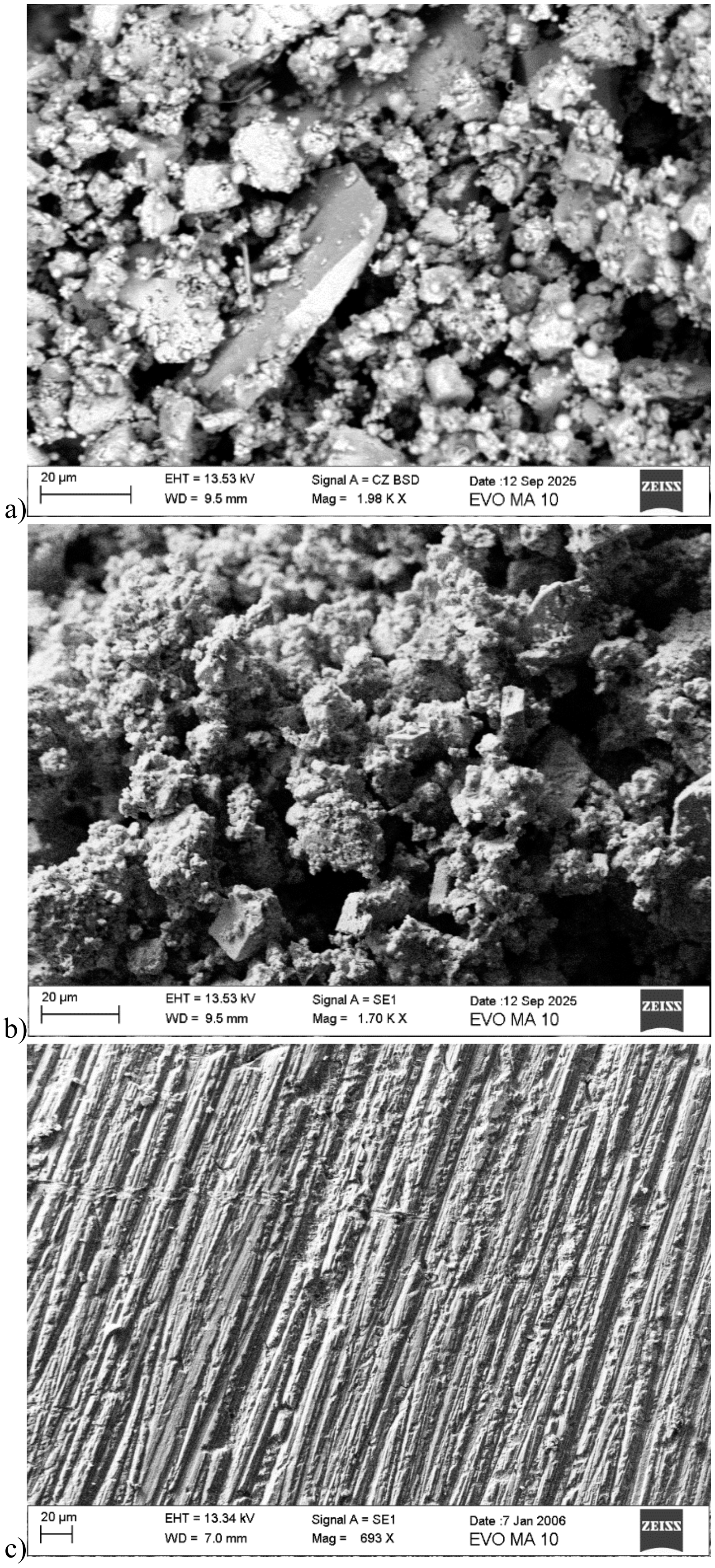

a)
b)
c)

Figure S2. SEM pictures of the $Sn_{38}Sb_8I_8$ samples: a) after ball-milling for 6 h, b) after ball-milling for 6 h and sintering at 620 K for 3 days, and c) after ball-milling for 6 h, sintering at 620 K for 3 days and hot pressing at 673 K and 80 MPa for 1 hour under Ar atmosphere.

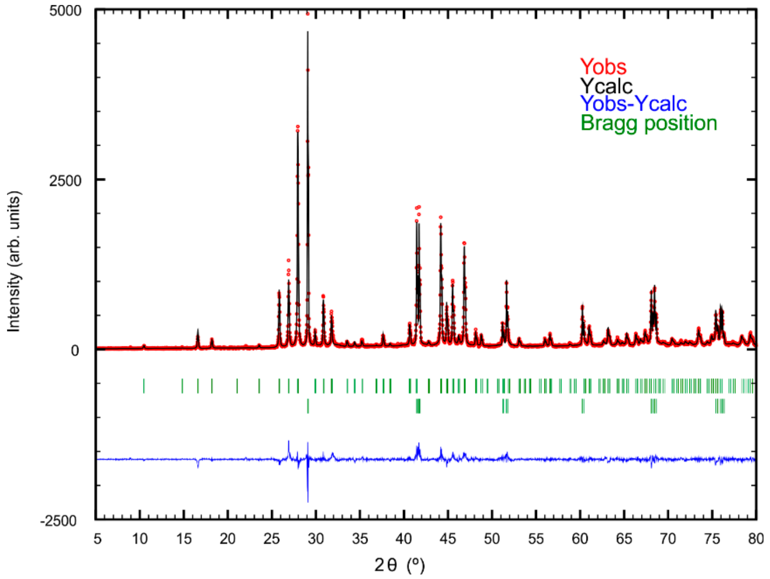

Figure S3. Rietveld plot using the powder X-ray diffraction data for the ball-milled and sintered $Sn_{30}Sb_{16}I_8$. Residual values: R_{wp} = 18.7%, χ^2 = 2.3, R_{Bragg}($Sn_{38}Sb_8I_8$) = 7.6%, R_{Bragg}(Sn-Sb alloy) = 7.4%. The top and bottom rows of Bragg peaks represent $Sn_{38}Sb_8I_8$ and a Sn–Sb alloy, respectively.

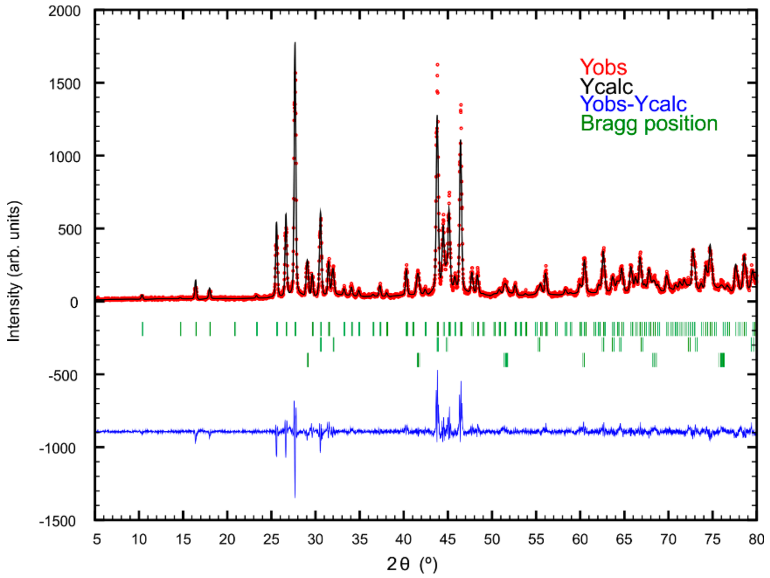

Figure S4. Rietveld plot using the powder X-ray diffraction data for the ball-milled, sintered and hot-pressed sample of $Sn_{38}Sb_8I_8$. Residual values: R_{wp} = 23.9%, χ^2 = 2.64, R_{Bragg}($Sn_{38}Sb_8I_8$) = 6.99%, R_{Bragg}(β-Sn) = 6.91%, R_{Bragg}(Sn-Sb alloy) = 10.8%. The top, middle and bottom rows of Bragg peaks represent $Sn_{38}Sb_8I_8$, β-Sn and a Sn–Sb alloy, respectively.
